# Growing a thriving international community for small-angle scattering through collaboration

**DOI:** 10.1107/S1600576721007561

**Published:** 2021-07-29

**Authors:** Jill Trewhella

**Affiliations:** a School of Life and Environmental Sciences, The University of Sydney, Building G08, Camperdown, NSW 2006, Australia

**Keywords:** small-angle scattering, SAS, triennial SAS conferences, International Union of Crystallography, IUCr, Guinier Prize, standards

## Abstract

Small-angle scattering emerged as a tool for studying noncrystalline structures from early observations around 1930 that there was a relationship between the extent of the scattering and the size of the scattering object. André Guinier, a leading figure in the development of the field, noted in his summary findings from the first Conference on Small Angle Scattering in 1958 that the technique would be of value to study ‘submicroscopical inhomogeneities’ and further provided a means of ‘observation [that had] in the past restricted the field of application of the X-ray method.’ In 1965 the first of what became a highly successful series of Small-Angle Scattering (SAS) meetings held approximately every three years took place in Syracuse, NY, USA, and many of these ongoing meetings published their proceedings and highlights in the International Union of Crystallography (IUCr) *Journal of Applied Crystallography*. Since the early 2000s, the relationship between the international SAS community represented at the triennial SAS meetings and the IUCr has been strengthened and deepened through formal cooperation and collaboration in a number of mutually beneficial activities that have supported the growth and health of the field and the IUCr.

## Abstract 2

This commentary describes the growth of small-angle scattering as a technique for structural characterization in noncrystalline systems across chemistry, biology and materials and how the international community continues to grow and prosper through highly successful international meetings and a strategic collaborative relationship with the International Union of Crystallography.

## 1. A brief history of an emerging small-angle scattering community   

The first published observations of small-angle X-ray scattering (SAXS) were in the *Indian Journal of Physics* by Panchapakesa Krishnamurti, whose series of papers from 1928 to 1930 culminated in a publication detailing the relationship between particle size and molecular weights and the extent of small-angle scattering (SAS) (Krishnamurti, 1930 ▸). In 1939, André Guinier published his landmark paper (Guinier, 1939 ▸) describing a simple approximate mathematical relationship between the rate of decrease in intensity of the SAS signal at the smallest angles and the size of the scattering particle. This relationship, known as the Guinier approximation, could be readily exploited for studying a broad range of materials and resulted in a significant uptick in applications of SAS. Guinier thus became widely known as the ‘father’ of small-angle scattering.

The influential monograph *Small-Angle Scattering of X-rays* co-authored by Guinier with Gérard Fournet (Guinier & Fournet, 1955 ▸), translated from the original French into English, provides a comprehensive review of the state of the field by the mid-1950s. The monograph covers the enabling theoretical frameworks, such as the Debye approximation (Debye, 1915 ▸) relating the total scattering from a particle to the scattering from volume elements within it, as well as advances facilitating laboratory measurements, such as the development of laboratory-based X-ray cameras with pin-hole or slit collimation, and further breakthroughs in data interpretation and analysis such as the Kratky plot (Kratky & Porod, 1949 ▸), used to assess flexibility in a scattering particle, and the Porod law (Porod, 1951 ▸) describing the high-angle scattering. The scattering of neutrons is also covered in the monograph, as researchers were beginning to consider how the distinctive properties of neutrons might be used to expand the opportunities for SAS. However, it would be a couple of decades before small-angle neutron scattering (SANS) would begin to grow, notably aided by the advocacy of Bernard Jacrot (Jacrot, 1976 ▸) and neutron scattering facilities at the Institut Laue–Langevin, Grenoble, France.

In 1958, the first conference dedicated to SAS was held in Kansas City, Missouri, USA (September 23–25), and the proceedings were published in the *Journal of Applied Physics* (*Small Angle X-ray Conference Proceedings*, Vol. 30, Issue 5, May 1959). Final summary remarks were contributed by André Guinier (Guinier, 1959 ▸), who notes ‘In conclusion, it can be said that the progress in our understanding of small angle scattering, as well as in other methods of observation have in the past restricted the field of application of the X-ray method, but we are now able to make a better use of this method as in cases where it is still the best tool to study submicroscopical inhomogeneities.’

In many ways, the 1958 conference could be considered the zeroth meeting for what would become the very successful series of meetings that would bring together the international SAS community across all fields to advance and grow SAS applications. It was just seven years on, in 1965, that what is regarded as the first of this series was held in Syracuse, New York, USA. Since then, the international SAS community has come together to meet approximately every three years (see Table 1 ▸). These triennial meetings are a major event for the field, consistently attracting >400 attendees since the 2006 Kyoto meeting. They highlight SAS contributions to biology, chemistry and materials science, much as Guinier envisioned with the technique becoming broadly applicable for materials characterization.

## 2. Growing a vibrant international community through collaboration with the International Union of Crystallography   

**Figure 1:**
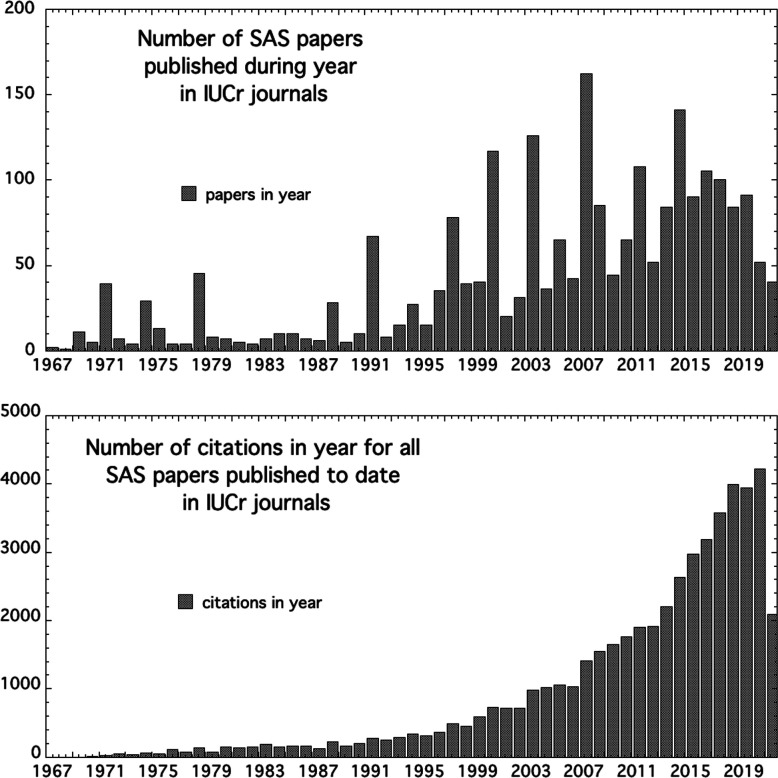
Small-angle scattering papers published in IUCr journals, and their associated citations (in all journals) from 1967 to mid-2021.

The early triennial SAS meetings were arranged on an *ad hoc* basis by successive host institutions whose representatives convinced the preceding conference delegates that they could do the job. In part because SAS at the time was viewed primarily in terms of being a sub-discipline within its application field, these meetings were organized independently from the International Union of Crystallography (IUCr). Even so, there was a strong IUCr connection that recognized SAS as a field of crystallography, with the publication of conference proceedings for many of the meetings in *Journal of Applied Crystallography* (Table 1 ▸). Further, IUCr journals have been a favoured destination for what are highly cited SAS studies (Fig. 1 ▸); the 2335 SAS papers published in IUCr journals since 1967 have generated 50 960 citations in 30 416 citing papers for an average citation per paper of 21.85.

In the early 2000s, Jan Skov Pedersen, John Barnes, Gernot Kostorz and other founding members of the IUCr Commission on Small-Angle Scattering (CSAS) saw value in greater collaboration between the IUCr and the triennial SAS meeting organizers. An agreement was negotiated wherein the international SAS community would adopt IUCr standards for speaker diversity and venue selection for the triennial SAS conference and the meeting would become an IUCr-sponsored meeting. That sponsorship would include support for students to attend and to enable participation from developing countries, as well as the establishment of the IUCr Guinier Prize of USD 5000, honouring the pioneering work of André Guinier. The Guinier Prize would be awarded for ‘lifetime achievement, a major breakthrough, or an outstanding contribution to the field of small angle scattering.’

As part of these negotiations, and in response to IUCr concerns to avoid scheduling clashes, the triennial SAS conference was moved out of the Congress years: hence the four-year break between the 2002 conference in Venice, Italy, and the 2006 conference in Kyoto, Japan (Table 1 ▸). Since then, successive CSAS Chairs have sought to solidify the connections between the IUCr and the triennial SAS meetings, in addition to supporting other independent SAS groups like canSAS (Collective Action for Nomadic Small Angle Scatterers; an ongoing activity to provide the small-angle scattering user community with shared tools and information http://www.cansas.org/) that have grown spontaneously from the SAS conference community.

## 3. A successful framework for sustaining meaningful collaboration   

An important mechanism to strengthen and sustain the IUCr–international SAS community connection has been to include IUCr CSAS members in processes for future conference venue selection and the selection of the Guinier Prize recipients. To this end, a formal process was established for venue selection for future meetings based on proposals submitted at least two meetings prior. The proposals are reviewed by representatives of the current triennial SAS meeting organizing committee members and members of CSAS. Qualified proposals based on a set of criteria agreed by the review committee (*e.g.* ensuring geographical diversity, programme committee diversity, local organizing capacity, adequate conference facilities, affordable accommodation, accessible food outlets, budgets with cost estimates *etc*.) are presented to the conference participants for a vote. In this way, the participants in the meeting still choose their future venue, but with the assurance that the proposed hosts are fully prepared for the challenging job they will have and with a full six years to prepare.

As with venue selection, nominations for the Guinier Prize are considered jointly by members of CSAS and members of the organizing committee for the meeting where the prize will be presented. It is often the case that the meeting will co-sponsor the prize by providing registration and travel support for the prize winner. The number of SAS luminaries who are Guinier Prize recipients (Table 2 ▸) is testimony to the success of the selection process, a credit to the field, and the prize constitutes a worthy recognition to the honouree.

In addition to the above formal mechanisms for collaboration on specific tasks, there are many ways in which CSAS and the triennial meeting organizers interact and exchange ideas, for example through engagement in the international advisory committee, programme and local organizing committees for the triennial meetings, and of course strong participation in the meetings. At the triennial SAS meetings, there is generally an open meeting of CSAS where the activities of CSAS are reported and there is opportunity for input to CSAS and exchange of ideas on initiatives. As the tenures of CSAS members tend to span multiple meetings, CSAS engagement offers some historical context and memory for what has worked well. The meeting organizing and programme committees also have been a source of excellent recruits to the membership of CSAS, with proven records for community engagement and effort.

Significantly, in 2020 when issues arose with the planned SAS2021 meeting in Campinas, Brazil, that would conflict with the COVID-19-postponed 25th IUCr Congress in Prague, the Chair of CSAS requested and was granted approval from the IUCr Executive for an additional CSAS member, specifically a representative of the SAS2021 meeting organizing committee. Through this mechanism, it was possible to work through the issues for both meetings with transparency and good will, which led to SAS2021 becoming SAS2022 with the 25th IUCr Congress moving to 2021 and the prospect of two successful meetings as the pandemic hopefully fades.

## 4. Examples of how the IUCr–SAS community collaboration advances science   

The SAS conferences and associated open CSAS meetings were important venues for discussion and dissemination of information by CSAS members who were leading efforts to establish a universal SAXS intensity standard (Allen *et al.*, 2017 ▸). The International Organization for Standardization (ISO) first published the *SRM 3600 – Absolute Intensity Calibration Standard for Small-Angle X-ray Scattering* ISO best practice measurement standard in 2015, and it was revised in 2020 (see https://www-s.nist.gov/srmors/view_detail.cfm?srm=3600). This standard was drafted in ISO/TC 24/SC4/WG10 where Technical Committee (TC) 24 is ‘Particle characterization including sieving’, sub-committee (SC) 4 is ‘Particle size characterization’ and Working Group (WG) 10 is ‘Small angle X-ray scattering method’. The same working group is now focused on surface area measurement.

Another example of the effectiveness of the collaborative relationship between the IUCr CSAS and the triennial SAS meeting organizers is the effort to establish community-endorsed standards for biomolecular SAS. Around the mid-1990s, biomolecular SAS evolved rapidly from a technique practiced predominantly by a select community of SAS experts to a tool for the broad structural biology community. From 1950 to 1995, biomolecular SAS accounted for 4% of the total publications using SAS, from 1996 to 2016 it was 12% and since 2017 it has been 18% (Web of Science Core Collection search on small-angle scattering and protein compared with small-angle scattering alone). This growth trajectory was the subject of considerable discussion at a meeting of CSAS in Kyoto at SAS2006, where several participants suggested that standards for the publication of biomolecular SAS data and models were needed. The topic was predictably controversial as there were legitimate concerns that efforts to have standards could have unintended consequences and restrict publication in ways that were not appropriate. With the seeds of this effort having been planted in Kyoto, however, there were many follow up discussions at subsequent triennial SAS meetings and IUCr Congresses. These discussions were expanded with the establishment of a SAS validation task force (SASvtf) (Trewhella *et al.*, 2013 ▸) by the world-wide Protein Data Bank (wwPDB). Eleven years after the discussions in Kyoto, guidelines for publication of biomolecular SAS studies, including standards for data presentation and model validation, were presented at the 24th IUCr Congress in Hyderabad, India (Trewhella & Guss, 2017 ▸), and published in *Acta Crystallographica Section D* (Trewhella *et al.*, 2017 ▸). These guidelines are being adopted, as shown by the 11 964 downloads (as of 23 July 2021) since going online.

Among the recommendations of the SASvtf and the 2017 publication guidelines was that biomolecular SAS data should be deposited in a public archive. Subsequently, the wwPDB Integrative/Hybrid Methods validation task force (IHMvtf) (Sali *et al.*, 2015 ▸) recommended that, for the new generation of biomolecular structures that use multiple types of data and computational methods, all the data types with their metadata should be publicly available in data archives. Furthermore, the data formats should facilitate seamless exchange between archives of different data types to support data and model validation. In response to these recommendations, there was further development of a SAS extension of the IUCr core Crystallographic Information File (sasCIF) (Kachala *et al.*, 2016 ▸; Malfois & Svergun, 2000 ▸), which was then utilized by the SAS Biological Data Base (SASBDB) (Valentini *et al.*, 2015 ▸) in establishing a searchable curated repository of experimental SAS data deposited together with the relevant experimental conditions and sample details. Because the SASBDB uses sasCIF, there can be seamless exchange with the wwPDB, and SAS was the first technique beyond crystallography, NMR and electron microscopy to be formally included in the wwPDB efforts to establish the capacity to accept structures determined using integrative/hybrid methods (Burley *et al.*, 2017 ▸; Berman *et al.*, 2018 ▸, 2019 ▸).

## 5. Looking to the future   

There are good reasons to continue to expand and deepen the connection between the IUCr and the international SAS community represented at the triennial meetings and at the IUCr Congresses.

The international SAS community continues to be very active in the IUCr Congresses, with CSAS sponsoring a keynote speech, five microsymposia (including a number co-sponsored with other commissions) and a special session (Advances in data and model validation in biomolecular small-angle scattering: impacts on data and meta-data recording and data archiving) for the upcoming 25th Congress (14–22 August 2021, Prague, Czech Republic). IUCr journals will continue to be a popular choice for leading SAS research, with an array of journals that are natural homes for SAS applications across chemistry, biology and materials, as well as for further technical developments in instrumentation, analysis and standards development. While the era of conventional conference proceedings in IUCr journals is effectively over, special issues of *Journal of Applied Crystallography* that collect up to 25 full-length high-quality peer-reviewed papers associated with work presented at the conference have worked well for both SAS2012 and SAS2015. It will be beneficial for each SAS conference organization to continue to work with IUCr journals to facilitate open access for such special issue papers.

The IUCr pioneered the development of standards, common data formats, open-access data and education in the field of crystallography, diffraction and scattering, and the SAS community represented at the triennial SAS meetings is very active in tackling these issues in their respective subdisciplines. Ongoing cooperation in these activities will be of great benefit, providing for knowledge transfer and collaboration and, importantly, the avoidance of multiple divergent efforts to achieve the same goal.

## Figures and Tables

**Table 1:** Small-angle scattering conferences and associated proceedings and special issues 1958–2020 The Roman numerals indicate the recognized triennial meetings, with the 1987 and 1958 meetings and their proceedings listed for completeness.

Sequence	Conference, location	No. attendees	Proceedings/special issues	No. papers, pages
XVII	SAS2018, Traverse City, MI, USA	411	–	–
XVI	SAS2015, Berlin, Germany	424	*J. Appl. Cryst.***49**, *Small-angle scattering special issue*, December 2016, highlighting contributions at SAS2015, bringing together articles originally published between October and December 2016	12 papers
XV	SAS2012, Sydney, Australia	426	*J. Appl. Cryst.***47**, *Small-angle scattering special issue*, February 2014, highlighting contributions at SAS2012, bringing together articles originally published between October 2013 and February 2014	25 papers
XIV	SAS2009, Oxford, UK	500	–	–
XIII	SAS2006, Kyoto, Japan	600	*J. Appl. Cryst.***40**, Part s1, April 2007	139 papers, 705 pages
XII	SAS2002, Venice, Italy	380	*J. Appl. Cryst.***36**, Part 3, Number 1, June 2003	69 papers, 319 pages
XI	SAS-99, Upton, NY, USA	310	*J. Appl. Cryst.***33**, Part 3, Number 1, June 2000	69 papers, 319 pages
X	SAS-96, Campinas, Brazil	210	*J. Appl. Cryst.***30**, Part 5, Number 2, October 1997	69 papers, 319 pages
IX	SAS-93, Saclay, Paris, France	190	*J. Phys. IV***3**, C8, 3–526 (1993)	109 papers, 523 pages
VIII	SAS-90, Leuven, Belgium	150	*J. Appl. Cryst.***24**, Part 5, October 1991	69 papers, 461 pages
–	1987, Argonne, Lemont, IL, USA	–	*J. Appl. Cryst.***21**, Part 6, December 1988	43 papers, 302 pages
VII	SAS-87, Prague, Czechoslovakia	120	*Macromol. Symp.***15**, 1–372 (1988)	372 pages
VI	SAS-83, Hamburg, Germany	100	–	–
V	SAS-80, Berlin, Germany	150	–	–
IV	SAS-77, Gatlinburg, TN, USA	140	*J. Appl. Cryst.***11**, Part 5, October 1978	71 papers and abstracts, 359 pages
III	SAS-73, Grenoble, France	70	*J. Appl. Cryst.***7**, Part 2, April 1974	58 papers and abstracts, 144 pages
II	SAS-70, Graz, Austria	65	*J. Appl. Cryst.***4**, 406–427 (1971)	64 extended abstracts, 21 pages
I	SAS-65, Syracuse, NY, USA	25	*Small-Angle X-ray Scattering* (edited by Harry Brumberger), Gordon & Breach Science Publishers, New York (1967)	32 papers, 509 pages
–	1958 Kansas City, MO, USA	–	*J. Appl. Phys.***30**(5), 601–674 (1959)	74 pages

**Table 2:** Guinier Prize recipients

2018	Dmitri Svergun (EMBL, Germany)
2015	Sow-Hsin Chen (MIT, USA)
2012	Otto Glatter (University of Graz, Austria)
2009	Vittorio Luzzati (Centre de Génétique Moléculaire, CNRS, Gif-sur-Yvette, France)
2006	Heinrich B. Stuhrmann (GKSS Forschungszentrum Geesthacht, Germany)
2002	Michael Agamalian (ORNL, Oak Ridge, TN, USA)
