# Growing a thriving international community for small-angle scattering through collaboration. Corrigendum

**DOI:** 10.1107/S160057672101164X

**Published:** 2021-11-16

**Authors:** Jill Trewhella

**Affiliations:** a School of Life and Environmental Sciences, The University of Sydney, Building G08, Camperdown, NSW 2006, Australia

**Keywords:** small-angle scattering, SAS, triennial SAS conferences, International Union of Crystallography, IUCr, Guinier Prize, standards

## Abstract

Errors in the article by Jill Trewhella [*J. Appl. Cryst*. (2021), **54**, 1029–1033] are corrected.

Table 1 in the article by Trewhella (2021 ▸) contains a number of errors. The SAS2009 conference proceedings, including 52 invited and contributed papers, were published in the *Journal of Physics – Conference Series*, Vol. 247, 2010. The correct numbers of papers and pages for SAS2002 should be 106 papers, 495 pages; for SAS-99 should be 105 papers, 445 pages; and for SAS-96 should be 63 papers, 327 pages.

In regard to the origins of canSAS, it was originally an initiative of staff at the Institut Laue–Langevin, European Synchrotron Radiation Facility (ESRF) and ESRF Collaborative Research Groups that came to be taken up by the broader SAS community with support at the SAS conferences.
